# Is There an Age Limit for a Trial of Vaginal Delivery in Nulliparous Women?

**DOI:** 10.3390/jcm12113620

**Published:** 2023-05-23

**Authors:** Gil Zeevi, Rita Zlatkin, Alyssa Hochberg, Shir Danieli-Gruber, Ohad Houri, Eran Hadar, Asnat Walfisch, Avital Wertheimer

**Affiliations:** 1 Helen Schneider Hospital for Women, Rabin Medical Center, Petach-Tikva 4941492, Israel; 2 The Sackler Faculty of Medicine, Tel Aviv University, Tel Aviv 6997505, Israel

**Keywords:** labor and delivery, pregnancy complications, advanced maternal age, cesarean section, elderly primipara, first vaginal delivery

## Abstract

Background: The number of nulliparous women over the age of 35 is consistently increasing, and the optimal delivery strategy is a subject of ongoing discussion. This study compares perinatal outcomes in nulliparous women aged ≥35 years undergoing a trial of labor (TOL) versus a planned cesarean delivery (CD). Methods: A retrospective cohort study including all nulliparous women ≥ 35 years who delivered a single term fetus at a single center between 2007–2019. We compared obstetric and perinatal outcomes according to mode of delivery—TOL versus a planned CD, in three different age groups: (1) 35–37 years, (2) 38–40 years, and (3) >40 years. Results: Out of 103,920 deliveries during the study period, 3034 women met the inclusion criteria. Of them, 1626 (53.59%) were 35–37 years old (group 1), 848 (27.95%) were 38–40 (group 2), and 560 (18.46%) were >40 years (group 3). TOL rates decreased as age increased: 87.7% in group 1, 79.3% in group 2, and 50.1% in group 3, *p* < 0.001. Rates of successful vaginal delivery were 83.4% in group 1, 79.0% in group 2, and 69.4% in group 3, *p* < 0.001). Neonatal outcomes were comparable between a TOL and a planned CD. Using multivariate logistic regression, maternal age was found to be independently associated with slightly increased odds for a failed TOL (aOR = 1.13, CI 95% 1.067–1.202). Conclusions: A TOL at advanced maternal age appears to be safe, with considerable success rates. As maternal age advances, there is a small additive risk of intrapartum CD.

## 1. Introduction

The number of nulliparous women over the age of 35 has consistently increased over the past four decades. In 2020, the mean maternal age at first delivery was 27.1 years, increasing from 21.4 years in 1970, and 11% of all first deliveries in nulliparous women in the United States were in women aged 35 years or older [1,2,3]. Studies have indicated that pregnancy at advanced maternal age is associated with increased risks of unfavorable maternal and neonatal outcomes. These include higher rates of preeclampsia, gestational diabetes, labor dystocia, emergency cesarean delivery (CD), post-partum hemorrhage, blood transfusion, low Apgar score, neonatal intensive care unit (NICU) admissions, and other unfavorable outcomes [4,5,6].

The optimum delivery strategy for this ever-growing population of elderly nulliparous women—a trial of labor (TOL) versus an elective CD—is a subject of ongoing discussion in studies and in everyday practice [6,7]. According to the most recent 2022 American College of Obstetricians and Gynecologists (ACOG) guidelines, vaginal delivery is regarded as safe and appropriate if there are no other maternal or fetal indications for cesarean delivery, and maternal age should not be an indication for elective CD [1]. Nevertheless, the rate of elective CD also increases with advanced maternal age, and there is a close correlation between maternal age and elective CD rates in a low-risk population [7]. CD confers an increased risk of maternal short- and long-term complications compared with a planned vaginal delivery, with prolonged recovery time, psychological effects, elevated risks for infection and thromboembolism, and an increased risk for perinatal complications in the subsequent pregnancy, including premature rupture of membranes, placental abruption, uterine rupture, placenta accreta, and neonatal asphyxia [8,9,10].

We therefore aimed to evaluate and compare obstetric and perinatal outcomes in nulliparous women aged ≥35 years undergoing a TOL versus a planned CD, and to assess success and complication rates in each of the groups.

## 2. Methods

### 2.1. Study Population

In this retrospective cohort study, we included all women aged 35 years and older who carried a singleton pregnancy and delivered a term fetus at Rabin Medical Center (RMC) between 2007 and 2019. RMC is a tertiary university-affiliated medical center.

Our tertiary university-affiliated medical center has one of the largest obstetrical units in Israel and is situated in a key metropolitan area, with over 8500 births annually and a 10% prevalence of emergency CD during a trial of labor.

The cohort was divided into 3 study groups of parturient women according to maternal age upon delivery. The first group consisted of women aged 35 to 37 years; the second group was aged 38 to 40; and the third group consisted of women aged 40 and older. We chose these age groups in order to accurately delineate perinatal complications specific to each age range, and our data enabled us to create three relatively large groups of patients according to this sub-division.

Each study group was further divided into women who underwent a TOL and women who did not opt for vaginal delivery and had an elective cesarean delivery (eCD).

Multiple pregnancies and pregnancies complicated by congenital anomalies or preterm deliveries were excluded.

### 2.2. Definitions

Advanced-age primigravida was defined as maternal age of over 35 years at the first delivery. Preterm birth was defined as delivery before 37 + 0 gestational weeks. Large-for-gestational-age (LGA) was defined as birthweight above the 90th percentile for gestational age, and small-for-gestational-age (SGA) was defined as birthweight below the 10th percentile for gestational age, according to the birthweight standards in the live-born population in Israel [11]. A low Apgar score was defined as less than 7 at either 1 min or 5 min assessments. Neonatal hypoxemia was defined as umbilical cord pH of less than 7.15.

The trial of labor group included women with either spontaneous or induced labor onset, with or without labor augmentation. Elective CD was mainly due to maternal request but also included parturients with an obstetrical diagnosis mandating CD, such as breech presentation, placenta previa, etc. In the TOL group, intrapartum CD indications included four categories: non-reassuring fetal heart rate (NRFHR), labor dystocia, failed induction of labor, and maternal request.

### 2.3. Data Collection

Data was retrieved from the RMC’s comprehensive computerized maternal and neonatal medical records, including records from the emergency room triage, delivery room, maternal–fetal hospitalization, and neonatal nursery or neonatal intensive care unit (NICU). Collected data included maternal demographics; medical and obstetrical background; antepartum, intrapartum, and postpartum complications; and mode of delivery, including CD indications.

Neonatal outcomes were also collected and included birthweight, 1 and 5 min Apgar scores, umbilical cord pH measurements, NICU admission, jaundice, need for phototherapy, hypoglycemia, intraventricular hemorrhage (IVH), neonatal respiratory complications, and neonatal death.

### 2.4. Outcome Measures

The primary outcome measure was the rate of successful vaginal deliveries. Secondary outcome measures included maternal and perinatal complications.

### 2.5. Statistical Analysis

Statistical analysis was performed using the SAS software (Version 9.4, SAS Institute, Cary, NC, USA). Comparisons between continuous variables were performed with Student’s *t*-test or Mann–Whitney U-test, and categorical data were compared using the χ^2^ test or Fisher’s exact test, as appropriate. A probability value of <0.05 was considered significant. Several multivariate logistic regression models were constructed in order to detect the association between maternal age and other risk factors for failed trial of labor and for adverse perinatal outcomes while controlling for possible confounders that were found to be significantly different between the groups on univariate analysis: induction of labor, pre-gestational and gestational diabetes mellitus, in vitro fertilization (IVF) pregnancy, pre-eclampsia, and gestational age at delivery.

### 2.6. Ethics

The study was approved by the Institutional Review Board of Rabin Medical Center (RMC 0264-20). Informed consent was waived owing to the study’s retrospective design.

## 3. Results

Out of 103,920 deliveries during the study period, 3427 (3.3%) women met the inclusion criteria. Of them, 1626 were between the ages of 35 and 37 years (group 1); 848 were between 38 and 40 years (group 2); and 560 were 40 years and older (group 3). In group 1, 1436 women (87.7%) underwent a TOL, and in groups 2 and 3, 3673 (79.3%) and 281 (50.1%) underwent a TOL, respectively.

The cohort’s baseline characteristics are presented in Table 1. In group 1, women who underwent a TOL were comparable to those who chose elective CD (eCD sub-group), with the exception of more IVF pregnancies (9.5% vs. 4.8%, *p* = 0.01) and higher rates of pre-gestational diabetes mellitus and preeclampsia with severe features in the elective CD sub-group (4.5% vs. 1%, *p* < 0.001; and 2% vs. 0.35%, *p* < 0.001, respectively). Group 2 presented similar trends, with higher rates of assisted reproductive technology (ART) and IVF pregnancies in the eCD sub-group (20.5% vs. 11.9%, *p* = 0.005, 21.4% vs. 7.5%, *p* < 0.001, respectively) and higher rates of preeclampsia with severe features (1.7% vs. 0.1%, *p* = 0.03). In group 3, women who did not pursue vaginal delivery were older (44.0 vs. 42.5 years, *p* < 0.001); had a higher gravidity range, indicating an increased rate of unsuccessful pregnancies; had higher rates of ART and IVF pregnancies (24.7% vs. 13.1% in the TOL group, *p* < 0.01, 41.2% vs. 20.6% in the TOL group, *p* < 0.001, respectively); conceived more frequently with egg donation (8.9% vs. 3.2%, *p* = 0.004); and had higher rates of preeclampsia without severe features (4.3% vs. 0.7%, *p* = 0.006).

We compared obstetric outcomes between the TOL and eCD subgroups in every age group (Table 2) separately. In all three groups, gestational age was higher in the TOL sub-group (groups 1 and 2: 39.2 vs. 38.2, *p* < 0.001; group 3: 39.1 vs. 38.0, *p* < 0.001). PPH rates were significantly higher in the TOL sub-group only in group 2 (3.8% vs. 0%, *p* = 0.003). However, a similar trend was found in groups 1 and 3, but without statistical significance (3.6% vs. 1%, *p* = 0.05 and 4.3% vs. 2.1%, *p* = 0.23 respectively). Maternal need for blood transfusion, placental abruption, and chorioamnionitis rates were comparable between the groups. There were no cases of intrauterine fetal demise (IUFD) or perinatal death in the entire cohort.

Neonatal outcomes are presented in Table 2. Birthweight was comparable between the eCD and TOL sub-groups in groups 1 and 3, while in group 2, the eCD sub-group had a slightly lower mean birthweight (3076.9 g vs. 3152.1 g, *p* < 0.001). Despite that, the rate of LGA newborns was higher in the eCD sub-group in all three age groups (12% vs. 2.8%, *p* < 0.001, 7.4% vs. 3.5%, *p* = 0.03, 5.4% vs. 2.1%, *p* = 0.04). There were no significant differences between the TOL and eCD sub-groups in any of the study groups in 1 and 5 minute Apgar scores, hypoglycemia, neonatal jaundice, respiratory complications, seizure incidence, IVH, NEC, and meconium aspiration rates. Higher rates of NICU admission were found in the TOL sub-group in group 1 (7.1% vs. 3.5%, *p* = 0.05), while in the eCD sub-group of group 1, we found higher rates of cord pH lower than 7.15 (10% vs. 5.4%, *p* = 0.003). No differences in these outcomes were found in any of the other study groups.

A post-hoc multivariate logistic regression analysis was used to manage potential variables for NICU admission. This analysis concluded that maternal age was not independently associated with NICU admission (Table 3). Induction of labor, pre-gestational and gestational diabetes mellitus, and TOL were all found to be independently associated with NICU admission.

Table 4 presents a comparison between women who underwent a TOL in the three study groups. The rate of successful vaginal delivery was over 69% in all study groups and highest in the younger group (83.4% in group 1, 79.0% in group 2, and 69.3% in group 3, *p* < 0.001).

Groups 1 and 2 were comparable in all parameters. However, differences were found between groups 1 and 2 and group 3. The rate of normal vaginal delivery and assisted vaginal delivery in group 3 was significantly lower (46.6% vs. 52.6% in group 2 and 58.2% in group 1, *p* < 0.001 for both, and 22.4% vs. 26% in group 2 and 24.6% in group 1, *p* < 0.001 for both, respectively). The intrapartum CD rate was highest in group 3 (30.6%) and lowest in group 1 (16.5%, *p* < 0.001). The most common indication for CD in all groups was NRFHR, and the least common indication was maternal request during labor. Group 3 had higher rates of NRFHR (9.9% vs. 8.4% in group 2 and 6.5% in group 1, *p* < 0.001 for both), labor dystocia (9.6% vs. 7.7% in group 2 and 7.1% in group 1, *p* < 0.001 for both), and failed induction (10.3% vs. 4.6% in group 2 and 2.8% in group 1, *p* < 0.001 for both). No differences were found between the three groups in induction of labor rates, perineal tears, or shoulder dystocia.

A post-hoc multivariate logistic regression analysis was utilized to assess the association between maternal age and mode of delivery (only among those who underwent a trial of labor, Table 5) while controlling for potential confounders, including labor induction, pre-gestational and gestational diabetes mellitus, and gestational age at delivery. Maternal age was found to be independently associated with slightly increased odds for failed TOL (aOR = 1.13, CI 95% 1.067–1.202).

## 4. Discussion

In this study, we aimed to evaluate the success rate of vaginal delivery and perinatal outcomes in nulliparous women aged 35 and above stratified by maternal age in order to improve our counseling and informed decision-making regarding the recommended mode of delivery in this unique and growing population. Our findings suggest that a trial of vaginal delivery at advanced maternal age is safe and holds high success rates.

As indicated by previous studies, first delivery at advanced maternal age has been associated with adverse maternal and neonatal outcomes compared to women younger than 35 years of age, including higher rates of CD instrumental vaginal delivery and NICU admission [12,13,14,15]. Consistent with our results, a study by Yogev et al., conducted at our institution [5], indicated a significant increase in CD rates according to the different age categories as follows: in nulli- and multiparous women, 16% between the ages of 20 and 29, 23% between the ages of 30 and 39, 43% between the ages of 40 and 44, and 78% above the age of 45.

Previous studies often compared advanced maternal age groups to non-advanced maternal age controls (below and above the age of 35); however, only a few studies examined the differential risks between different advanced maternal age subgroups [14,16,17]. In the present study, we compared the differential risks across different subgroups of advanced maternal age nulliparous women, to precisely define the perinatal complications specific to each age range.

Based on our results, increasing maternal age was associated with higher rates of failed trial of labor, but after controlling for potential confounders, this increase was relatively small (aOR = 1.13), and women over the age of 40 demonstrated a successful TOL rate of over 69%, with no apparent major increase in maternal or neonatal adverse outcomes. A possible explanation for the higher rate of CD in our older age group might be physician inclination to opt more easily towards a CD during labor in patients perceived as less likely to conceive and deliver again in the future. As this was a retrospective study, we recognize that such inherent bias could not be controlled for.

In line with our results, Schwartz et al. found a 74% success rate in a TOL among nulliparous and multiparous women ≥ 50 years of age and underlined that the main predictor of outcome is maternal health and not maternal age [18].

Bayrampour et al. demonstrated, in their systematic review, that deliveries in advanced maternal age increased the risk of both elective and emergency CD with ORs between 1.39 and 2.76, and Jacquemyn Y et al. from Belgium presented a significant linear increase of primary CD with increasing age, which is consistent with our results (25–34 years (8.9%), 35–39 years (15.2%), 40–44 years (17.8%), and 45 years and older (27.3%)) [19,20].

In our study, we did not find significant differences in obstetric and neonatal outcomes between the TOL and eCD sub-groups, except for higher rates of PPH in the TOL group with no differences in the need for maternal blood transfusion, and an increased rate of LGA newborns in the eCD group, which can be associated with a greater incidence of pre-gestational diabetes mellitus in this group, and may operate as a potential co-factor for CD indication.

The TOL sub-group aged 35–37 had a higher rate of NICU admission, but after adjusting for potential confounders, maternal age was no longer a significant predictor for this outcome. This finding might be related to the higher rate of birth asphyxia, meconium aspiration, and hypoxic-ischemic encephalopathy in vaginal delivery compared to eCD [21]. Contrarily, the eCD sub-group aged 35–37 had a higher rate of cord pH < 7.15. Given the increased risk that eCD infants may experience respiratory distress and the need for additional treatments such as mechanical ventilation, surfactant administration, or nitric oxide inhalation [21]. All this information should be taken into consideration when consulting regarding mode of delivery in this population.

The two main predictors of NICU admission, according to our research, are TOL and induction of labor. This finding may be attributed to the increased rates of instrumental vaginal delivery in this group and the inherent risks of vaginal birth itself, and it suggests that maternal age is not an independent risk factor for NICU admission.

Induction of labor was found to be the primary independent risk factor leading to a failed TOL, with an increased risk of intrapartum CD due to failed induction as maternal age increases. This finding is consistent with earlier research. Claramonte Nieto and colleagues reported that maternal age ≥ 40 years was associated with an increased risk of CD after induction compared with younger women, mainly because of failed induction but with no association to other adverse maternal or neonatal outcomes, and according to Bergholt et al., the risk of CD increased three to five times for every 5 year increase in a woman’s age [19,20,22,23,24,25]. In contrast, the Walker et al. RCT and the ARRIVE trial found that inducing labor in low-risk, nulliparous patients at 39 weeks reduced the risk of CD. However, there are some differences between our study group and the cohort in these trials. Walker’s RCT included women who were over 35, which is comparable to our cohort as opposed to the 4–5% in the ARRIVE study. Additionally, the cesarean section rates in the ARRIVE trial (19–23%) and Walker’s trial (32–33%) are significantly higher than the percentage of primary CD at our institution [26,27].

It is essential to stress that the first cesarean delivery’s repercussions are not innocent, with more short- and long-term complications compared with a planned vaginal delivery, including a longer recovery period, psychological effects, increased risks for infection, thromboembolism, and bleeding, and a longer hospital stay [9]. Bi et al. demonstrated that maternal age ≥ 35 years at first CD is a risk factor for premature rupture of membranes, placental abruption, uterine rupture, and neonatal asphyxia in the following pregnancy [10]. In the Danish National Birth Cohort, Jackson S. et al. evaluated the morbidity following primary CD and discovered an elevated risk in the subsequent pregnancy for anemia, placental abruption, uterine rupture, and hysterectomy [28,29].

These findings emphasize that a planned CD due to maternal request or caregiver’s advice due to advanced age should be appropriately discussed in this population, with all aspects taken into consideration, including fetal safety and maternal morbidity [1,9,10,28]. Our study suggests that a TOL is a relatively safe option in this population, without increased risk for maternal or neonatal complications that are age-dependent, except for a higher intrapartum CD rate.

The strength of this study lies in the fact that it was performed at a single large tertiary center that follows uniform guidelines. Additionally, the number of cases included in each group is relatively large, with a well-stratified cohort of age groups over 35, all delivering at term. Our study is not free of limitations, mainly due to its retrospective nature and the associated inherent biases. Data regarding body mass index (BMI), the indication for labor induction, and Bishop score upon induction, as well as outcome data regarding younger parturients (<35 years), were missing. Additionally, due to our division of patients into groups with a relatively narrow age range, it is possible that our sample sizes were underpowered to detect smaller differences and rarer outcomes between groups.

In conclusion, although advanced maternal age is associated with decreased TOL success rates, it appears to be safe and reasonable in primiparous women aged 35 years and above. Maternal age, per se, should not be considered an indication for eCD.

## Figures and Tables

**Table 1:** Baseline characteristics of the study population.

	35–37 Age Group *n* = 1626			38–40 Age Group *n* = 848			>40 Age Group *n* = 560		
	Trial of Labor	No Trial of Labor	*p*-Value	Trial of Labor	No Trial of Labor	*p*-Value	Trial of Labor	No Trial of Labor	*p*-Value
	*n* = 1426	*n* = 200		*n* = 673	*n* = 175		*n* = 281	*n* = 279	
Age, years	35.84 (±0.8)	35.81 (±0.8)	0.59	38.8 (±0.8)	38.9 (±0.8)	0.123	42.5 (±1.8)	44.0 (±2.7)	<0.001
Gravidity	1 (1–6)	1 (1–7)	0.7	1 (1–6)	1 (1–6)	0.1	1 (1–7)	1 (1–14)	0.01
ART	98 (6.8%)	22 (11%)	0.07	80 (11.9%)	36 (20.5%)	0.005	37 (13.1%)	69 (24.7%)	<0.001
IVF	69 (4.8%)	19 (9.5%)	0.01	51(7.5%)	37 (21.4%)	<0.001	58 (20.6%)	115 (41.2%)	<0.001
Egg Donation	0 (0%)	1 (0.5%)	0.123	3 (0.45%)	2 (1.1%)	0.2	9 (3.2%)	25 (8.9%)	0.004
Hypertensive disorders									
Gestational HTN	20 (1.4%)	1 (0.5%)	0.5	16 (2.3%)	0	0.03	5 (1.7%)	4 (1.4%)	1
Chronic HTN	10 (0.7%)	0	0.62	13 (1.9%)	3 (1.7%)	1	1 (0.3%)	3 (1%)	0.36
Mild PET	20 (1.4%)	2 (1%)	1	8 (1.1%)	6 (3.4%)	0.08	2 (0.7%)	12 (4.3%)	0.006
Severe PET/HELLP	5 (0.35%)	4 (2%)	<0.001	3 (1.7%)	1 (0.1%)	0.03	5 (1.78%)	3 (1.08%)	0.7
Pregestational Diabetes	15 (1%)	9 (4.5%)	<0.001	9 (1.3%)	5 (2.8%)	0.1	5 (1.7%)	8 (2.8%)	0.4
Gestational Diabetes	171 (11.9%)	33 (16.5%)	0.08	78 (11.6%)	29 (16.5%)	0.09	44 (15.6%)	56 (20.0%)	0.1
Oligohydramnios	65 (4.5%)	5 (2.5%)	0.26	22 (3.2%)	3 (1.7%)	0.32	10 (3.5%)	3 (1.08%)	0.08
Polyhydramnios	14 (0.9%)	3 (1.5%)	0.45	3 (0.45%)	0	1	3 (1.07%)	4 (1.4%)	0.72

Values are presented as mean ± standard deviation or median (range) for continuous variables and as *n* (%) for categorical variables. Abbreviations: ART—assisted reproductive technologies; IVF—in vitro fertilization; HTN—hypertension; PET—preeclampsia; HELLP—hemolysis, elevated liver enzymes and low platelet count.

**Table 2:** Obstetric and Perinatal outcomes.

	35–37 Age Group *n* = 1626			38–40 Age Group *n* = 848			>40 Age Group *n* = 560		
	Trial of Labor	No Trial of Labor	*p*-Value	Trial of Labor	No Trial of Labor	*p*-Value	Trial of labor	No Trial of Labor	*p*-Value
	*n* = 1426	*n* = 200		*n* = 673	*n* = 175		*n* = 281	*n* = 279	
Gestational age at delivery, weeks	39.2 (±1.2)	38.2 (±0.9)	<0.001	39.2 (±1.26)	38.2 (±0.95)	<0.001	39.1 (±1.2)	38.0 (±0.9)	<0.001
Birth weight, grams	3163.7 (±424.7)	3206.9 (±539.1)	0.09	3152.1 (±424)	3076.9 (±494)	<0.001	3142.4 (±303)	3122.0 (±419)	0.3
Epidural anesthesia	1125 (78.8%)	168 (84%)	0.15	541 (80.3%)	143 (81.7%)	0.56	227 (80.7%)	228 (81.7%)	0.39
General anesthesia	46 (3.2%)	13 (6.5%)	<0.001	13 (4.6%)	25 (8.9%)	<0.001	13 (4.0%)	31 (8.8%)	<0.001
Manual lysis	50 (3.5%)	0 (0%)	0.003	18 (2.67%)	0	0.033	18 (6.4%)	1 (0.3%)	<0.001
PPH	51 (3.58%)	2 (1%)	0.055	26 (3.8%)	0	0.003	12 (4.27%)	6 (2.15%)	0.23
Maternal blood transfusion	6(0.4%)	0	1	2 (0.3%)	0	1	2 (0.7%)	1 (0.3%)	1
Placental abruption	8 (0.56%)	0 (0%)	0.6	5 (0.74%)	0 (0%)	0.59	2 (0.6%)	1 (0.3%)	0.5
SGA	53 (3.72%)	10 (5%)	0.43	34 (5.0%)	10 (5.7%)	0.7	10 (3.5%)	6 (2.1%)	0.44
LGA	40 (2.8%)	24 (12%)	<0.001	24 (3.57%)	13 (7.4%)	0.03	6 (2.1%)	15 (5.38%)	0.04
1-min Apgar < 7	58 (4.07%)	4 (2%)	0.17	32 (4.7%)	8 (4.5%)	1	9 (3.2%)	6 (2.1%)	0.6
5-min Apgar < 7	9 (0.6%)	0	0.61	9 (1.3%)	1 (0.5%)	0.69	2(0.7%)	0	0.5
Cord pH < 7.15	77 (5.4%)	20 (10%)	0.003	70 (10.4%)	12 (6.8%)	0.43	20 (7.1%)	21 (7.5%)	0.74
NICU admission	102 (7.15%)	7 (3.5%)	0.05	55 (8.1%)	8 (4.5%)	0.1	21 (7.4%)	14 (5%)	0.29
TTN	20 (1.4%)	3 (1.5%)	0.7	10 (1.5%)	1 (0.5%)	0.4	4 (1.4%)	1 (0.3%)	0.37
RDS	3 (0.18%)	0	1	3 (0.45%)	0	1	1 (0.3%)	3 (1/08%)	0.37
Asphyxia	29 (2.0%)	0	0.04	16 (2.38%)	4 (2.3%)	1	7 (2.5%)	3 (1.08%)	0.33
Seizures	4 (0.28%)	0 (%)	1	3 (0.45%)	2 (1.1%)	0.2	1 (0.3%)	0	1
Neonatal hypoglycemia	9 (0.6%)	3 (1.5%)	0.17	8 (1.2%)	2 (1.1%)	1	3 (1.07%)	3 (1.08%)	1
Meconium aspiration	4 (0.28%)	0 (0%)	1	4 (0.6%)	0	0.58	1 (0.3%)	0	1
Neonatal death	2 (0.14%)	0 (0%)	1	0 (0%)	0 (0%)		1 (0.3%)	1 (0.3%)	1

Values are presented as mean±standard deviation or median (range) for continuous variables and as *n* (%) for categorical variables. Abbreviations: PPH—postpartum hemorrhage; NICU—neonatal intensive care unit; SGA—small for gestational age; LGA—large for gestational age; TTN—transient tachypnea of the newborn; RDS—respiratory distress syndrome.

**Table 3:** Multivariate logistic regression model for NICU admission.

	Adjusted Odds Ratio	95% Confidence Interval	*p*-Value
Maternal age	1.005	1.072–0.942	0.8798
Assisted reproductive technologies	0.593	1.275–0.932	0.0117
Induction of labor	1.892	3.278–1.092	0.0229
Without pregestational or gestational diabetes	0.478	0.778–0.293	0.003
Preeclampsia	0.677	1.673–0.274	0.3985
Week of delivery	1.093	1.275–0.937	0.2595
Elective cesarean delivery	0.474	0.822–0.273	0.0079

**Table 4:** Delivery outcomes in women who underwent a trial of labor.

	35–37 Age Group	38–40 Age Group	>40 Age Group	*p*-Value (35–37 vs. 38–40)	*p*-Value (38–40 vs. >40)	*p*-Value (35–37 vs. >40)
	*n* = 1426	*n* = 673	*n* = 281			
Induction of labor	511 (35.8%)	235 (34.9%)	109 (33.8%)	0.8	1	1
Vaginal delivery	1190 (83.4%)	532 (79%)	195(69%)			
Mode of delivery				0.07	0.048	<0.001
Normal vaginal delivery	830 (58.2%)	354 (52.6%)	131 (46.6%)			
Vacuum extraction	352 (24.7%)	175 (26%)	63 (22.4%)			
Forceps delivery	8 (0.56%)	3 (0.4%)	1 (0.36%)			
Perineal tear grade 3/4	8 (0.49%)	3 (0.4.5%)	2 (0.7%)	1	0.67	0.67
Cervical tear	6 (0.37%)	3 (0.4.5%)	0 (0%)	1	0.59	0.59
Shoulder dystocia	2 (0.12%)	3 (0.4.5%)	0 (0%)	0.18	1	1
Cesarean delivery	236 (16.6%)	141 (20.95%)	86 (30.6%)	0.07	<0.001	<0.001
Cesarean indication						
Fetal distress	93 (6.5%)	57 (8.4%)	28 (9.9%)	0.31	<0.001	<0.001
Labor dystocia	102 (7.1%)	52 (7.7%)	27 (9.6%)			
Failed induction	41 (2.8%)	31 (4.6%)	29 (10.3%)			
Maternal request	0 (0%)	1 (0.15%)	0 (0%)	0.31	1	1

Values are presented as *n* (%) for categorical variables.

**Table 5:** Multivariate logistic regression model for a failed trial of labor.

	Adjusted Odds Ratio	95% Confidence Interval	*p*-Value
Maternal age	1.13	1.067–1.202	<0.001
Assisted reproductive technologies	1.26	0.870–1.846	0.21
Induction of labor	2.1	1.50–2.98	<0.001
Pregestational and gestational diabetes	1.72	1.085–2.79	0.02
Preeclampsia	1.23	0.48–3.15	0.65
Gestational age (weeks)	1.31	1.158–1.498	<0.001

## Data Availability

Data available on request due to restrictions eg privacy or ethical. The data presented in this study are available on request from the corresponding author.
